# Transcriptomic signatures of schizophrenia revealed by dopamine perturbation in an ex vivo model

**DOI:** 10.1038/s41398-018-0216-5

**Published:** 2018-08-16

**Authors:** Jubao Duan, Harald H. H. Göring, Alan R. Sanders, Winton Moy, Jessica Freda, Eugene I. Drigalenko, Mark Kos, Deli He, Pablo V. Gejman, MGS, P. V. Gejman, A. R. Sanders, J. Duan, D. F. Levinson, J. Shi, N. G. Buccola, B. J. Mowry, R. Freedman, A. Olincy, F. Amin, D. W. Black, J. M. Silverman, W. F. Byerley, C. R. Cloninger, D. M. Svrakic

**Affiliations:** 1 Center for Psychiatric Genetics, NorthShore University HealthSystem, Evanston, IL USA; 2 Department of Psychiatry and Behavioral Neuroscience, University of Chicago, Chicago, IL USA; 3 South Texas Diabetes and Obesity Institute, University of Texas Rio Grande Valley School of Medicine, San Antonio, TX USA; 4 Department of Genetics, Texas Biomedical Research Institute, San Antonio, TX USA; 5 NorthShore University HealthSystem and University of Chicago, Chicago, IL USA; 6 Stanford University, Stanford, CA USA; 7 National Cancer Institute, Rockville, MD USA; 8 Louisiana State University Health Sciences Center, New Orleans, LA USA; 9 Queensland Centre for Mental Health Research, Brisbane and Queensland Brain Institute, The University of Queensland, Brisbane, Australia; 10 University of Colorado, Denver, CO USA; 11 Atlanta Veterans Affairs Medical Center and Emory University, Atlanta, GA USA; 12 University of Iowa Carver College of Medicine, Iowa, IA USA; 13 Mount Sinai School of Medicine, New York, NY USA; 14 University of California, San Francisco, CA USA; 15 Washington University, St. Louis, MO USA

## Abstract

The dopaminergic hypothesis of schizophrenia (SZ) postulates that dopaminergic over activity causes psychosis, a central feature of SZ, based on the observation that blocking dopamine (DA) improves psychotic symptoms. DA is known to have both receptor- and non-receptor-mediated effects, including oxidative mechanisms that lead to apoptosis. The role of DA-mediated oxidative processes in SZ has been little studied. Here, we have used a cell perturbation approach and measured transcriptomic profiles by RNAseq to study the effect of DA exposure on transcription in B-cell transformed lymphoblastoid cell lines (LCLs) from 514 SZ cases and 690 controls. We found that DA had widespread effects on both cell growth and gene expression in LCLs. Overall, 1455 genes showed statistically significant differential DA response in SZ cases and controls. This set of differentially expressed genes is enriched for brain expression and for functions related to immune processes and apoptosis, suggesting that DA may play a role in SZ pathogenesis through modulating those systems. Moreover, we observed a non-significant enrichment of genes near genome-wide significant SZ loci and with genes spanned by SZ-associated copy number variants (CNVs), which suggests convergent pathogenic mechanisms detected by both genetic association and gene expression. The study suggests a novel role of DA in the biological processes of immune and apoptosis that may be relevant to SZ pathogenesis. Furthermore, our results show the utility of pathophysiologically relevant perturbation experiments to investigate the biology of complex mental disorders.

## Introduction

Schizophrenia (SZ) is a severe brain disorder with 0.5–1% prevalence and ~80% heritability estimated from twin studies^1^. The primary evidence for the hyperdopaminergic hypothesis of SZ rests on the observation that psychotogenic stimulants such as methamphetamines lead to elevated brain dopamine (DA) levels and can cause psychosis^2–10^. Furthermore, brain imaging studies have shown that amphetamine-induced increases in DA response are correlated with positive symptoms of SZ (e.g., hallucinations and delusions)^11–13^. Recent SZ genome-wide association studies (GWAS) suggest an association between common variants at the *dopamine receptor D2* (*DRD2)* locus and SZ^14^. However, DA is also known to have non-receptor mediated functions, e.g., through DA autoxidation upon exposure to air or oxygen, a process that contributes to DA neuron loss in Parkinson’s disease (PD) and other neurodegenerative disorders involving DA neurotransmission^15,16^.

While classical hypotheses of SZ etiology focus on neuronal origins of the disease, the strongest and most replicable SZ-GWAS finding is at the extended major histocompatibility complex (xMHC) region^14,17–21^ that is known to contain many genes playing important roles in the immune system. Most recently, common alleles of the xMHC region immune system gene, *complement component 4A* (*C4A*), have been shown to be able to explain much of the xMHC region GWAS signal, and *C4A* showed increased expression in SZ postmortem brains^22^. Even without considering the xMHC region, GWAS-implicated genes are also enriched in key immune processes such as TGF-β signaling, B-cell activation, and T-cell activation^14^. Cytokines play roles in cytotoxicity and apoptosis, as well as influencing DA and other neurotransmitter systems^23,24^ that are implicated in the pathophysiology of SZ. Anti-inflammatory agents, such as celecoxib and aspirin, are reported to ameliorate some psychotic symptoms^23^, and classic antipsychotics affect microglial cells and astrocytes in the central nervous system (CNS) partly through the modulation of the expression of *cyclo-oxygenase-2* (*COX-2*)^25,26^. There is also growing evidence from clinical studies with COX-2 inhibitors that points to favorable effects of anti-inflammatory therapy in SZ^25,26^. Most recently, type I interferon (IFN) was shown to activate the synapse pruning function of microglia in lupus-prone mice, suggesting a mechanism underlying the prevalent neuropsychiatric conditions in patients with systemic lupus erythematosus^27^.

We hypothesized that some pathogenic effects of DA may be mediated through non-receptor mechanisms, and that some of these effects may be detectable in cells from a primary immune tissue. Lymphoblastoid cell lines (LCL) collections are the most accessible and sizable samples available for functional studies. Given that a large proportion of gene expression signatures are shared between different tissues^28–35^, we have used LCLs as a model to gain insights on SZ biology^36–38^. Here, we have analyzed RNAseq data of 1204 LCLs derived from 514 European-ancestry (EA) SZ cases and 690 controls in the absence or presence of DA, and have identified different effects of DA on transcriptomic profiles between SZ cases and controls.

## Materials and methods

### Samples

The initially processed RNAseq sample consisted of 515 SZ cases and 692 controls, and the final analyzed sample contained 514 SZ cases and 690 controls after excluding three expression outliers (see QC below). These EA subjects are from the GWAS- and CNV-studied portion of the MGS collection^17,20,39^ and have previously been reported on for their transcriptomics only at the baseline (unstimulated) condition^37^. There are 639 males (263 cases and 376 controls) and 565 females (251 cases and 314 controls). Detailed phenotypic data have been previously described^40^. Accession numbers from the database of Genotypes and Phenotypes (dbGaP) include phs000775, phs000021, and phs000167. The NorthShore University HealthSystem Institutional Review Board approved the study.

### Cell culture and RNA preparation

LCLs of the study sample were derived at Rutgers University Cell and DNA Repository (RUCDR)^36^. For each LCL, we measured EBV (viral) load (copy number), viable cell count (to index growth rate), and ATP level (to index energy status) at cell harvest (for use as covariates in expression analyses), which are known to have an effect on gene expression in LCLs^41^. For the initially processed 515 SZ cases and 692 controls, RNAseq was carried out in five large batches; further detailed methodology was previously described^37^. For DA perturbation (the pilot on four LCLs and the large-scale RNAseq samples), we grew cells in independent wells (on 6-well plates) in the presence or absence of DA at indicated concentrations. DA perturbation lasted 24 h. To block DA effects, we pre-treated the cells with the DA receptor antagonists for 6 h before adding DA to the cell culture medium. These DA blockers included: D1-like receptor (D1 or D5) antagonist SCH23390 (200 nM; ~100-fold saturation concentration^42^) and D2-like receptor (D2, D3, or D4) antagonist spiperone (200 nM; ~100-fold saturation concentration^43^). We purchased DA, SCH23390, and spiperone from Sigma-Aldrich. We included batch as a possible confounding variable in the analysis, i.e., as a covariate.

### RNAseq and data processing

RNA sequencing was carried out at the University of Minnesota Genomics Center (UMGC) on an Illumina HiSeq2000 at a depth of ~10 M reads/sample. RNAseq data for baseline and DA-stimulated samples were processed as previously described^37,38^. We aligned the 50-bp single reads to the human reference gene map (Gencode v20) using the mapping tool Tophat v2.0.5^44^, allowing for two mismatches. We counted the raw reads by using the HTseq-count script (www-huber.embl.de/users/anders/HTSeq/doc/overview.html)^45^ and calculated gene level expression as RPKM^46^ based on the exon model of the longest transcript of a gene (Gencode v20). We then quantile-normalized gene level RPKMs to help account for batch/run bias^38^.

### Quality control

For RNAseq data quality control (QC), we examined the mean Pearson correlations of gene level RPKM among 10 technical replicates (i.e., same RNA) and among 16 biological replicates (independent cell cultures of the same LCL)^38^. To capture genes with possible changes from or to very-low expression values upon DA stimulation, we analyzed all the genes (*N* = 21,043) with RPKM > 0 in at least 50% of either baseline or DA-stimulated samples. All samples included in the RNAseq had > 6 M mappable reads. We performed initial sample QC by: (1) checking for consistency between expression levels of chromosome X (*XIST*) and chromosome Y genes (*RPS4Y1*, *ZFY*, *USP9Y*, *DDX3Y*, *UTY*, *KDM5D*, and *EIF1AY*) vs. reported sex, and (2) by comparing RNAseq-called genotypes (using SAMtools mpileup function^47^, requiring > 8 reads at a called SNP site) with previous GWAS SNP genotypes (Affymetrix 6.0)^17,20^ for a panel of 175 informative SNPs^17,20^. The initial sample QC left us with 515 SZ cases and 692 controls. We carried out additional QC analyses to assure no systematic expression bias to either cases or controls, no batch effects, and to identify any potential outlier samples. We first compared the percentage of cases and controls that express each gene (i.e., sample completion rate) at baseline or DA stimulation condition (Fig. S1A-B). Under both baseline and DA-stimulation conditions, the sample completion rate (i.e., proportion of samples with RPKM > 0) in SZ cases and controls were highly correlated (*R* = 0.99; Fig. S1). Genes with higher sample completion rate differences between DA and baseline conditions tended to have lower expression values. However, such sample completion rate differences in cases and in controls were highly correlated (*R* = 0.91 for all the tested genes, and *R* = 0.98 for the subset of genes tested for SZ-associated differential DA response; Fig. S1). Furthermore, the DA-induced gene expression FCs in cases and controls are also well matched (i.e., did not show significant bias to either cases or controls; Fig. S2). Comparing expression correlations among all samples further identified two samples appearing to be “outliers” under the DA stimulation condition (Fig. S3A-B). The same two outliers were also among the three outliers identified by expression PCA (Fig. S3C-D). Both cases and controls appeared to be evenly distributed in a single big cluster in all the plots of expression PCs, except for those outlier samples (Fig. S3C-D). After excluding those three outliers (1 case and 2 controls), a total of 514 SZ cases and 690 controls remained for DA-perturbed differential gene expression analyses.

### Differential gene expression analyses

We first tested differential gene expression before and after DA stimulation. To capture genes that showed very different expression levels in the absence or presence of DA, we considered those with RPKM > 0 in ≥ 50% of baseline and/or DA-stimulated samples as expressed genes (*N* = 21,043). The rationale of using a more relaxed expression cut-off (50%) here vs. a previously used cut-off (80%)^38^ was to capture genes that might be expressed at a very-low level under either baseline or DA-stimulated condition, but potentially elevated in the other condition. For both baseline and DA-stimulated expression data, we regressed out the possible effects of affection status, sex, age, cell counts, EBV load, ATP level, genotypic ancestry principal components (PCs 1–5), and sequencing batch (5 batches). Although the cell counts and ATP levels at baseline conditions were just moderately correlated with the same measurements under DA stimulation condition (*R* = 0.60), we included the cell counts and ATP levels at both conditions in the regression analyses, which actually gave very similar results to the analysis when only baseline cell counts and ATP levels were regressed out (Fig. S4). The residuals were used in a paired Student’s *t*-test to identify genes showing differential expression between baseline and DA-stimulated conditions. FC for a gene was calculated as the ratio of its gene expression value in the presence of DA vs. baseline. The significance of the differential expression was FDR adjusted.

To identify genes that differ in expression level between SZ cases and controls (at either baseline or after DA stimulation), we first log2 (RPKM value + 1)-transformed the expression values, and then used standard multiple linear regression analysis to regress out all the above-listed covariates (except for affection status). The resulting residuals were rank-normalized (to ensure a normal distribution), and are the covariate-adjusted normalized expression values at baseline and upon DA stimulation. We then performed a single linear regression analysis of SZ status (independent variable) on these residuals (dependent variable) to identify the genes whose expression differs between cases and controls at either baseline or upon DA perturbation. The linear regression model is (E(rank-normalized residualized expression of gene X) = beta_0 + beta_1 × affection status + epsilon). The same approach, but with one additional step, was used to identify genes showing differential DA response between SZ cases and controls. Namely, we subtracted the covariate-adjusted normalized expression values at baseline from the DA state, to obtain the covariate-adjusted expression response of DA perturbation. Subsequently, affection status was regressed against these response variables, using a single linear regression model as above. We also explored the effect of regressing out the top 5 expression PCs, after already regressing out the previously mentioned known covariates, in the linear regression analyses, as a check for analytical robustness, in particular the possibility that the selected measured covariates may not have adequately captured all potentially existing confounder effects, which very well might be tagged by top PCs.

### Gene ontology and gene set enrichment analyses

We used the DAVID tool^48^ and WebGestalt^49^ for GO-term enrichment analyses, with all the genes expressed in LCLs as the background gene set. REVIGO^50^ was used to cluster and visualize the enriched GO-terms. Because of the large number of genes showing differential expression upon DA stimulation (>90%; Fig. 3), for GO-term enrichment analysis we selected a subset of genes that showed relatively larger magnitudes presumably representing more likely meaningful biological changes. Based on the distribution of all FCs, we used an arbitrary cut-off of 1SD and included all the genes with FCs of <0.88 or >1.23 (*N* = 3756), representing ~20% expression changes upon DA stimulation. For SZ-associated differentially expressed genes, we used FDR < 5% to select genes for GO-term enrichment analyses. We used all the LCL-expressed genes in our data set as a reference gene list for enrichment analyses.

For enrichment analysis of specific gene sets, we assembled different gene sets relevant to SZ pathogenesis based on public databases and literature searches as we described^38^, including 14,295 adult brain-expressed genes accounting for ~81% of well-annotated protein-coding genes^51^, 227 SZ-CNV genes within the 17 SZ-associated CNVs^39,52–55^, 435 genes with SNPs (within 500 kb) showing genome-wide significant (GWS) association to SZ in the Psychiatric Genomics Consortium (PGC2) sample^14^, and 863 genes that were differentially expressed (*P* < 0.05) between SZ cases and controls in the Stanley Array Collection of postmortem frontal cortex samples (www.stanleygenomics.org) (Table S8). For genes showing SZ-associated differential DA response, or differential expression at baseline or in the DA-stimulated condition, we counted the number of genes in each gene set. Using all the LCL-expressed genes in each gene set as the denominator, we then estimated the enrichment of each category of differential expressed genes in a pre-assembled gene set (Table 1). We evaluated the significance of the gene set enrichment using Fisher’s exact test (two-sided).

**Table 1:** Gene set enrichment among genes showing differential DA response, differentially expressed in baseline or in DA-stimulated LCLs between SZ cases and controls (FDR < 5%)

Analysis	Gene group	Total	Adult brain	% (*p*-value)	PGC2 GWS	% (*p*-value)	SZ-CNV	% (*p*-value)	Stanley_DEG	% (*p*-value)
	All genes	21,043	10,829	51.46%	297	1.41%	145	0.69%	407	1.93%
Regressing out known coavariates	Differential baseline expr	6581	4508	68.50% (3.7E-133)	113	1.72% (0.080)	36	0.55% (0.255)	174	2.64% (6.7E-04)
	Differential expr with DA	4507	3065	68.01% (2.4E-60)	75	1.66% (0.042)	23	0.51% (0.222)	122	2.71% (3.7E-05)
	Differential DA response	1455	1145	78.69% (3.2E-96)	25	1.72% (0.360)	14	0.96% (0.254)	50	3.44% (3.3E-04)
Regressing out known coavariates + top 5 PCs	Differential baseline expr	561	316	56.33% (0.023)	11	1.96% (0.275)	2	0.36% (0.596)	15	2.67% (0.210)
	Differential expr with DA	542	301	55.54% (0.061)	8	1.48% (0.853)	3	0.55% (0.241)	7	1.29% (0.340)
	Differential DA response	588	461	78.40% (2.4E-40)	15	2.55% (0.033)	5	0.85% (0.608)	23	3.91% (0.002)

RNAseq data set is from 1204 LCLs (514 MGS SZ cases and 690 controls) in the absence (baseline) or presence of 100 μM DA (DA stimulated). Linear regression was used for the analyses, either regressing out known covariates or also regressing out the top 5 expression PCs (principal components). Differential DA response was computed on baseline and dopamine residuals. For gene set enrichment analysis, genes are classified as brain expressed (*N* = 14,295; ~81% of well-annotated genes) in adult brain^51^, genes with PGC2 SZ genome-wide significant (GWS) SNPs within 500 kb of the gene (*N* = 436), genes (*N* = 107) in the 17 SZ-risk CNVs^55^, and genes that are differentially expressed between SZ cases and controls of Stanley Array Collection of postmortem frontal cortex samples (Stanley_DEG; *N* = 863, *p* < 0.05). Two-sided Fisher’s Exact test is used to estimate the significance of enrichment (*p*-values were shown under the % of genes), and the ones shaded with gray were significant after multiple testing correction

## Results

**Fig. 1:**
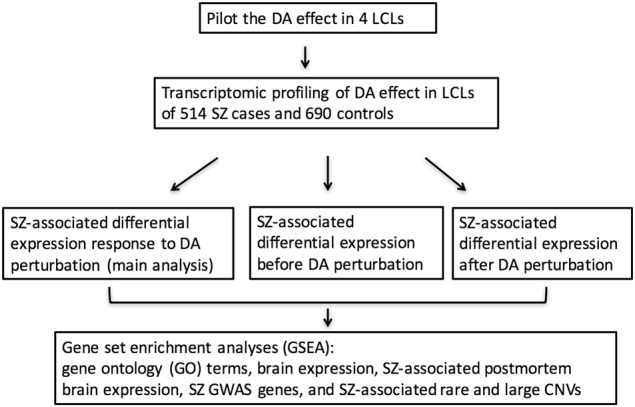
Flow chart of the study design. SZ schizophrenia, GWAS genome-wide association study, CNV copy number variation, DA dopamine, LCL lymphoblastoid cell line

To investigate the impact of DA exposure on LCLs, we first explored which concentration of DA showed robust effects in LCLs. Subsequently, we examined the transcriptomic profiles associated with DA exposure to identify expression responses associated with SZ (Fig. 1).

### DA effects on LCL growth and gene expression

**Fig. 2:**
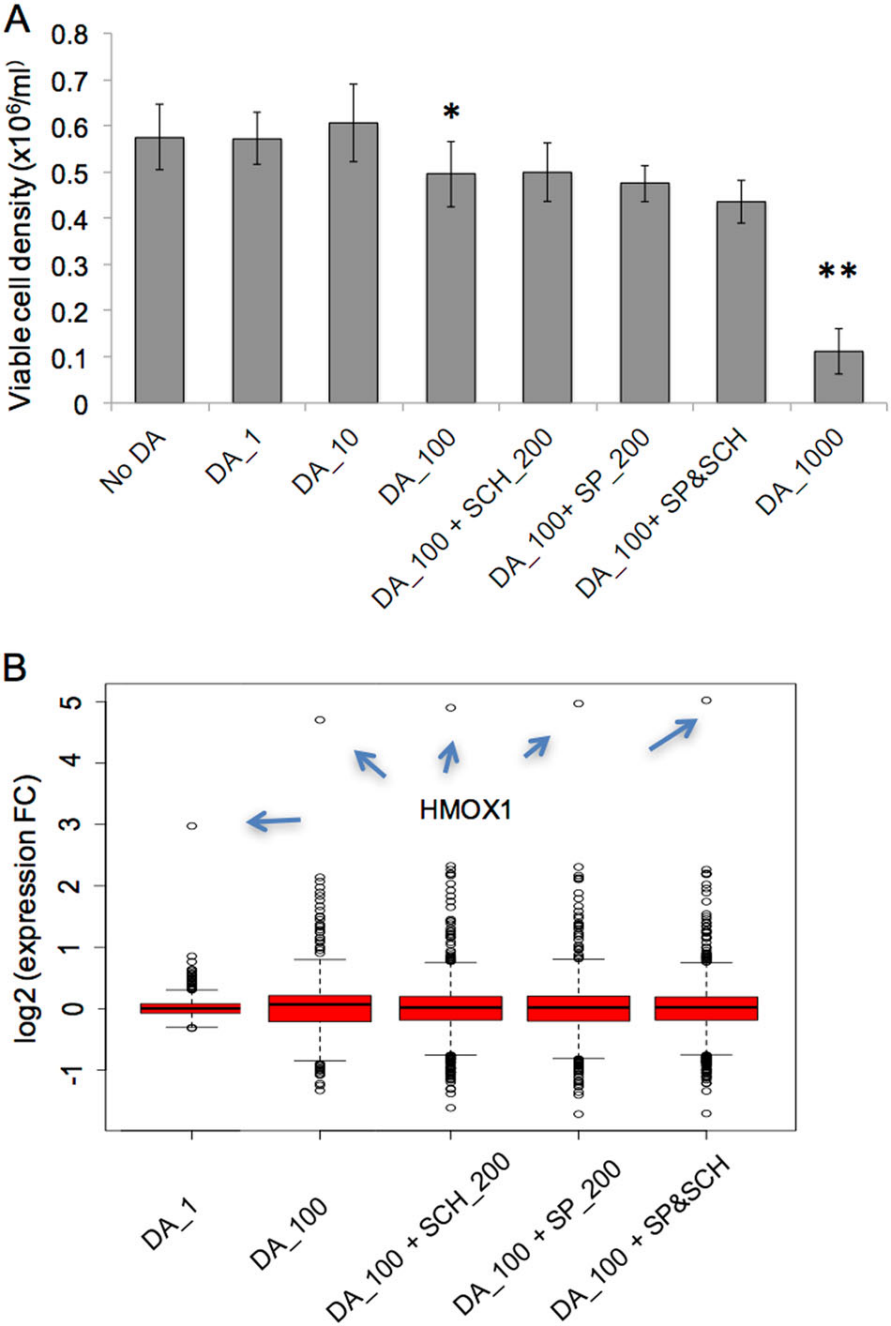
Dosage effect of dopamine (DA) on cell growth and genome-wide expression of 4 LCLs. **a** DA effect on cell growth after 24 h of DA treatment (1 μM, 10 μM, 100 μM, and 1,000 μM). 100 μM DA started to show a significant inhibitory effect on cell growth, and most cells were dead at 1000 μM DA. Pre-treatment of the cells with the D1 receptor antagonist SCH23390 (200 nM) and/or the D2 receptor antagonist Spiperone (SP; 200 nM) for 6 h did not block the inhibitory effect of DA on cell growth. * *P* < 0.05 and ** *P* < 0.01 were derived from two-sided paired Student’s *t*-test. **b** Box plot of DA effect on genome-wide expression. 100 μM DA showed a substantially stronger effect than 1 μM DA, but pre-treatment of the cells with D1 or D2 receptor antagonists did not reverse the effect of DA. Plotted are 2999 genes that showed significant expression changes after DA stimulation (100 μM) (*P* < 0.05). Arrows point to the values for *HMOX1*

We tested different DA concentrations in four LCLs of control subjects. We found that DA, at a commonly used concentration range of 100–150 μM^56–58^, started to show an inhibitory effect on LCL growth by ~20% (no effect with smaller DA concentrations) (Fig. 2a), an effect probably related to an apoptotic effects of DA^59^. At higher concentrations (1000 μM DA), which is near the estimated DA concentration (1.6 mM) in rat brain synaptic cleft^60^, ~80% of the LCLs were dead after 24 h of exposure (Fig. 2a). Pre-treating LCLs with DA receptor antagonists before applying DA did not block the overall DA effect (Fig. 2b), which was consistent with our observation that LCLs did not show significant expression of DA receptors (Reads Per Kilobase of transcript per Million mapped reads, RPKM < 0.1) in LCLs. In the baseline condition, 23,966 genes were expressed in all four LCLs (RPKM > 0). Approximately 13% (*n* = 2,999) of the genes showed significant expression changes in the presence of 100 μM DA (nominal *P* < 0.05; paired Student’s *t*-test). As expected, a lower DA concentration (1 μM) showed a substantially smaller magnitude of gene expression changes (Fig. 2b). As above, the DA effects were not blocked with DA receptor antagonists (Table S1). We decided to use a DA concentration of 100 μM in our DA perturbation experiment with a large sample, since this concentration achieved a widespread impact on gene expression with limited cell death.

**Fig. 3:**
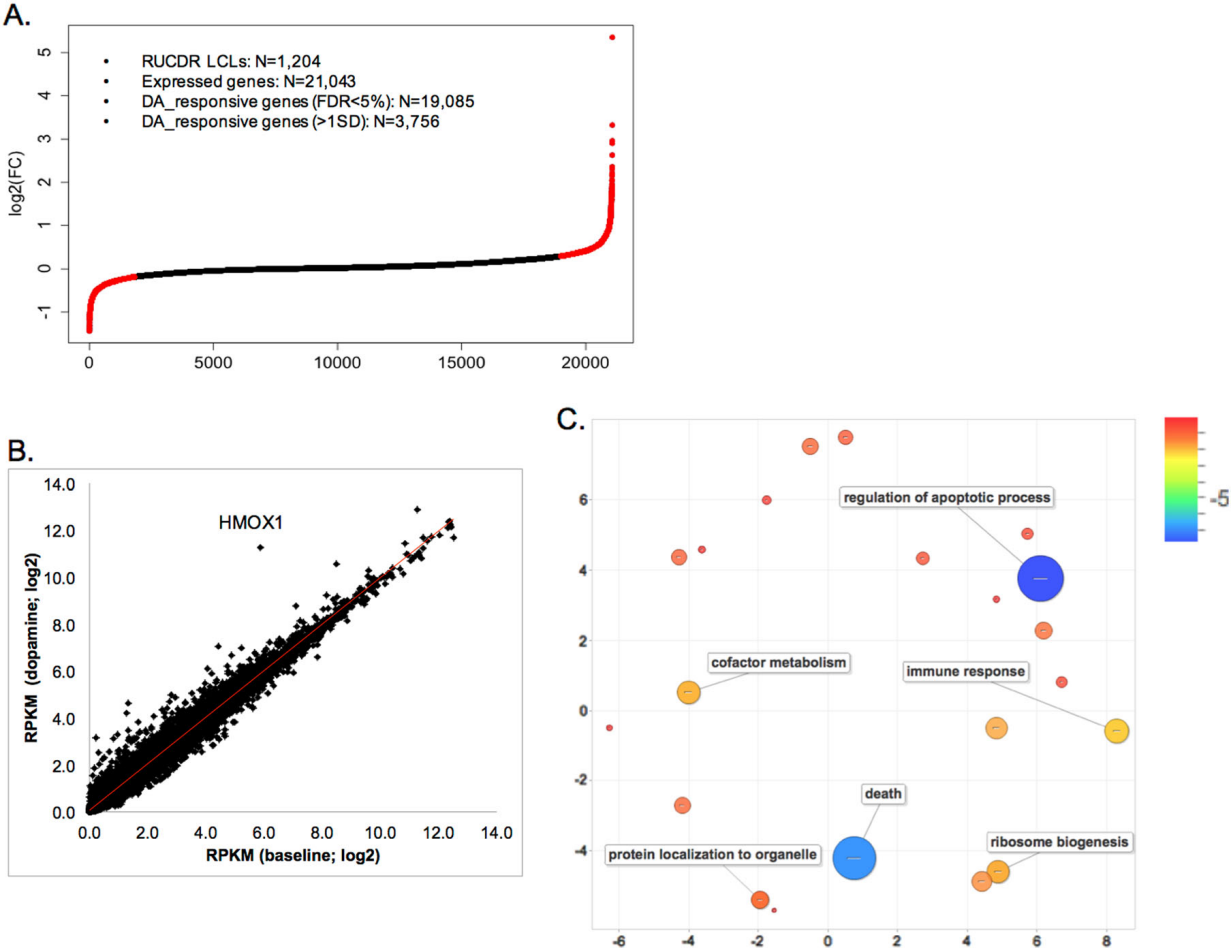
DA-responsive genes in 1204 LCLs of MGS subjects. **a** Distribution of expression fold change (FC) for all genes expressed in LCLs upon DA (100 μM) stimulation. An expressed gene was defined as having RPKM > 0 in at least 50% of baseline or DA-stimulated samples. Two-side paired Student’s *t*-test was used to test for DA response. The red dots show values for genes with log2 FC beyond the 1 SD cut off. **b** Gene expression before and after DA stimulation are highly correlated (Pearson *R* = 0.993). **c** GO-term enrichment analysis by DAVID. The enriched GO-terms were clustered and visualized by REVIGO^50^. Scale bar = log(FDR), blue indicates the most significant enrichment

We next studied the gene expression profiles of 1207 LCLs (515 MGS SZ cases and 692 controls) in the absence (baseline) or presence (DA-stimulated) of 100 μM DA. A total of 21,043 genes were expressed (RPKM > 0) in at least 50% of either baseline or DA-stimulated samples. We found no differences in the proportion of case and control samples expressing a given gene (Fig. S1) or for the expression fold change (FC) upon DA stimulation (Fig. S2). We also identified three samples that were outliers which we removed from further analyses (Fig. S3). The analysis of 1204 clean samples (514 SZ cases and 690 controls) showed that ~91% (19,085) of all the expressed genes were responsive to DA (FDR < 5%; paired Student’s *t*-test; Fig. 3a and Table S2). The FCs were small in most of these genes (Fig. 3a), but 150 genes showed > 2-fold expression differences (Table S2). Gene expression was very strongly correlated between the baseline and DA-stimulated conditions (*R* = 0.993; Fig. 3b). To better understand the biology of DA-responsive genes, we carried out gene ontology (GO) term enrichment analysis for 3756 genes with expression FCs > 1SD (1SD cut-off represents ~20% expression change upon DA stimulation). About 1762 genes showed reduced expression and 1994 showed increased expression. GO-terms related to apoptosis and immune responses were enriched (Fig. 3c). The most enriched GO-terms in the 1994 DA-upregulated genes were related to apoptosis, while the most enriched GO-terms in the 1762 DA-downregulated genes were related to RNA processing and metabolism (Table S3). These results are in agreement with the hypothesis that DA stimulation had a widespread toxic effect leading to cell death and apoptosis, as previously described^59^.

We examined whether genes showing relatively large FC were related to DA effects in the brain. Out of the five genes (*HMOX1*, *GDF15*, *AMBP*, *SLC48A1*, and *NQO1*) showing > 5-fold expression changes (see Table S3), four were expressed at highly elevated levels (*HMOX1*, *GDF15*, *AMBP*, and *NQO1*) and have brain functions likely related to DA mechanisms. The most stimulated (>42-fold) gene, *HMOX1 (heme oxygenase 1)*, is hypothesized to induce pathological brain iron sequestration under oxidative stress^61^ and has been reported to be upregulated by DA in cultured rat astrocytes, where astrocyte-specific proteins and pathways (e.g., impairment of glutamate transporters) play important roles in neurodegenerative diseases^62^. Furthermore, transgenic mice overexpressing *HMOX1* showed SZ-relevant features including increased tyrosine hydroxylase (TH), augmented DA and serotonin levels in basal ganglia, and attenuated prepulse inhibition^63^. Interestingly, over-expression of *HMOX1* in brain has been reported in Alzheimer’s disease (AD), PD, multiple sclerosis (MS), and other degenerative and nondegenerative CNS diseases^61,64,65^. The second most stimulated (10-fold) gene, *GDF15 (**growth differentiation factor 15**)*, encodes a trophic factor for midbrain DA neurons^66,67^. *AMBP* (*alpha-1-microglobulin/bikunin precursor*) has been proposed to be a urinary marker for major depression^68^, and *NQO1 (**NAD(P)H dehydrogenase, quinone 1**)* encodes an enzyme which removes quinone, leading to protection of DA cells^69–71^. *NQO1* has also been considered as a SZ candidate gene^72^, although unsupported by SZ-GWAS^14^. These results strongly suggest that the non-receptor mediated DA effects in LCLs may be relevant to brain disorders including SZ.

### DA-mediated transcriptomic responses associated with SZ

We hypothesized that some DA-induced transcriptomic changes would differ in SZ cases and controls and used multiple linear regression analysis to test whether DA-induced expression FC was associated with disease status. Cell count and ATP level were correlated at both baseline and DA-stimulated conditions (R = 0.58 for both variables). We also examined analytical robustness by repeating the analysis omitting the cell counts and ATP levels obtained after DA stimulation as covariates, which yielded very similar results (Fig. S4). Overall, 1455 genes (~7% of all analyzed genes) showed SZ-associated differential DA response at FDR < 5% (Table S2). Among the top-ranking genes were *interferon-induced protein with tetratricopeptide repeats 3* (*IFIT3*; rank #2) and *interferon receptor 1* (*IFNAR1*; rank #15; Table S2), implying a possible pathogenic role of IFN signaling genes. Interestingly, *IFIT3* and *IFNAR1* have been recently implicated in blood transcriptome profiles of recurrent major depression^73^. Also among the top-ranking genes showing SZ-associated differential DA response was *tumor necrosis factor receptor superfamily, member 11b* (*TNFRSF11b*; rank #1), which may be involved in TNF-α-induced apoptosis^24^. The most enriched GO-terms among genes showing SZ-associated differential expression response to DA stimulation were immune system process and response (FDR < 1.1–1.3 × 10^–11^) as well as response to virus (FDR < 1.99×10^−8^; Table S5). GO-terms related to regulation of apoptosis were also highly enriched (FDR < 3.41 × 10^−5^; Table S5). Since it is possible that the chosen covariates did not sufficiently tag all existing confounders, we further carried out an exploratory analysis where we regressed out the effects of the top five expression PCs after accounting for the effects of the above-mentioned covariates. In spite of this analysis substantially reducing the total number of SZ-associated differential DA-responsive genes (from 1455 to 588; Table S2), the top-ranked genes and the most enriched GO-terms remained very similar (Table S6). Together with the observed inhibitory effect of DA on LCL growth, these results suggest that certain immune genes, including those related to apoptosis, may play a role in SZ pathogenesis and mediate the differential response to DA between cases and controls.

We then tested whether the set of genes showing differential DA response in SZ was enriched for genes that were: (1) brain expressed, (2) differentially expressed in schizophrenia postmortem brains, (3) loci showing GWS association to SZ, or (4) located in SZ-risk CNVs^55^. We found significant enrichment of brain-expressed genes (1.5-fold enrichment; *P* = 3.2 × 10^−96^, two-sided Fisher’s exact test). In the Stanley SZ postmortem brain collection (www.stanleygenomics.org) we observed enrichment of SZ-associated genes (1.8-fold enrichment; *P* = 3.3 × 10^−4^, two-sided Fisher’s exact test; Table 1). Furthermore, we found a non-significant enrichment of genes spanned by SZ-associated CNVs^55^ (1.4-fold enrichment; *P* = 0.25, two-sided Fisher’s Exact test; Table 1). We also observed a nominally significant enrichment of SZ-GWAS genes (1.8-fold enrichment, *P* = 0.03, two-sided Fisher’s Exact test; Table 1). Table S7 shows the 25 SZ-GWAS genes and 8 SZ-CNV genes that showed differential response to DA between SZ cases and controls. These genes include *dihydropyrimidine dehydrogenase* (*DPYD*) at 1p21.3, one of the loci showing the strongest association with SZ, although the strongest association signal there clusters around two microRNAs^14^, MIR137 and MIR2682, where we have found a rare functional enhancer SNP possibly associated with both SZ and bipolar disorder^74^. Four genes (*MAPK3, SPN*, *TAOK2*, and *YPEL3*) out of the 8 SZ-CNV genes that showed SZ-associated differential expression response to DA are in the 16p11.2 duplication CNV, suggesting a possible role for DA dysfunction in the pathogenic mechanism underlying this CNV’s association with SZ (Table S7).

### Comparison of case-control gene expression differences in baseline and DA-stimulated conditions

**Fig. 4:**
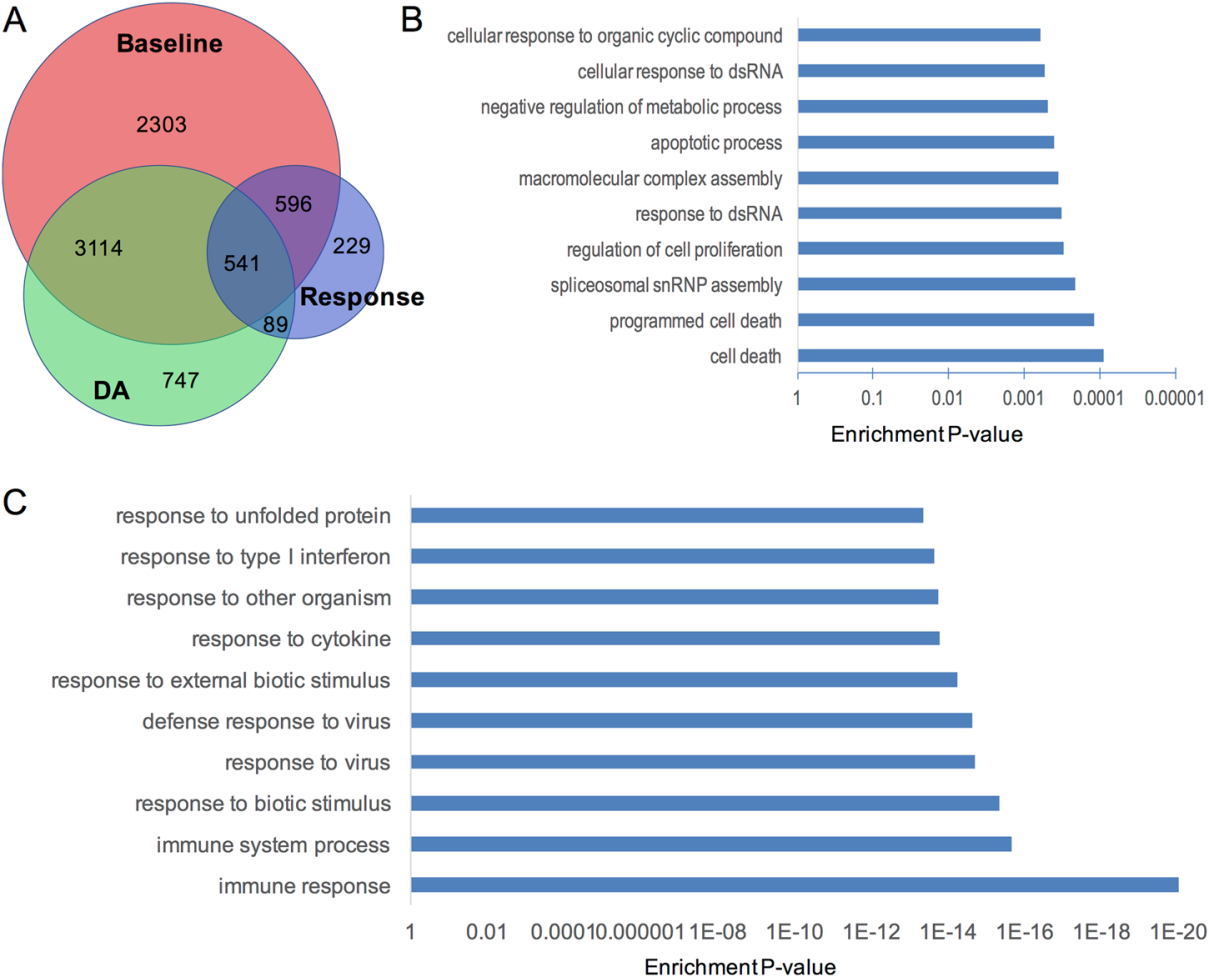
Genes showing SZ-associated differential expression under different conditions and the enriched gene ontology terms. **a** Venn diagram that shows the overlap of genes showing SZ-associated differential DA response, differentially expressed genes in baseline and under DA stimulation. **b** 229 genes that only showed SZ-associated differential response to DA are highly enriched for GO-terms related to cell death. **c** 541 genes that showed SZ-associated differential expression in all three analyses are highly enriched for GO-terms related to immune response and response to virus

We have recently reported genes that showed differential expression at the baseline (unstimulated) condition between SZ cases and controls in a meta-analysis of both RNAseq (complete overlap with this current study) and microarray data sets with similar sample size^37^. Here we examined whether genes showing SZ-associated differential DA response also showed case-control differential expression under baseline or DA-perturbed conditions. Using a similar linear regression analysis as described above, we tested the case-control gene expression differences at baseline and upon DA stimulation, respectively. When a pre-defined set of cell culture and demographic covariates was regressed out, we found that the SZ-associated differentially expressed genes under both conditions showed a substantial overlap (*n* = 3655; Fig. 4a and Table S2), and the directions of case/control differential expression for the overlapping genes were completely concordant (except for one RNA gene ENSG00000278514.1) (Fig. S5). About 84% of the genes showing SZ-associated differential DA response were also differentially expressed at baseline and/or DA-stimulation conditions (Fig. 4a). Genes that only showed SZ-associated differential DA response (but did not differ at either baseline or after DA exposure) are most enriched for GO-terms related to apoptosis (Fig. 4b), while the rest are most enriched for GO-terms related to immune response (Fig. 4c).

In our alternative model using both known covariates and the top five expression PCs, geared towards making sure that we adjust even for unknown potential confounders, we found a substantially smaller number of genes that showed SZ-associated differential expression under each condition (Table 1 and Fig. S6). Most (92%) of the genes showing SZ-associated differential DA response under this analytical model were not differentially expressed at either baseline or DA-stimulation condition (Fig. S6). Similar to the genes showing SZ-associated differential DA response, genes that were differentially expressed in SZ cases under baseline or DA-perturbed conditions are also highly enriched for GO-terms involving immune response.

Enrichment analyses of baseline and DA-stimulated conditions yielded overlapping but different gene sets (Fig. 4). We found that differentially expressed genes in cases in the baseline condition (but not upon DA stimulation) showed a nominally significant enrichment for PGC2 SZ GWS genes (*P* = 0.04, two-sided Fisher’s Exact test). Furthermore, compared to genes differentially expressed in case/control in the baseline condition, genes showing SZ-associated differential DA response showed more fold enrichment of brain-expressed genes (1.5 vs. 1.3), SZ-CNV genes (1.4 vs. 0.8) and Stanley SZ-associated brain-expressed genes (1.8 vs. 1.4; Table 1). Interestingly, in an exploratory analysis where both known covariates and the top 5 expression PCs were regressed out, only genes showing SZ-associated differential DA response showed significant enrichment for brain-expressed genes and Stanley SZ-associated brain-expressed genes (Table 1). Finally, five SZ-CNV genes and four SZ-GWAS genes that showed SZ-associated differential DA response did not show SZ-associated differential expression under the baseline condition (Table S7). Our results suggest that DA perturbation was instrumental to detect relevant transcriptomic profiles missed by the analysis of baseline condition only.

## Discussion

SZ-risk loci include both common and rare variants in many genes, each with small to modest effects on disease risk, as well as polygenic contributions of individually even smaller, and so far impossible to localize, effects^75,76^. We have studied the differential transcriptomic effects of DA perturbation of LCLs from SZ cases and controls, and found that a substantial number of genes responded to DA perturbation, that the DA response differed for many genes between SZ cases and controls, and that these differential response genes are enriched for immune and apoptosis-related genes, as well as those implicated by previous SZ-GWAS and CNV studies. Most genes showing SZ-associated differential expression under DA stimulation were also differentially expressed at baseline conditions (Figs. 3a & 4). However, 16% of the genes showing differential response to DA by SZ case-control status did not show differential expression at the baseline condition. This suggests that relevant but cryptic biological mechanisms associated with schizophrenia become detectable in our model only by functional perturbation.

Although abnormal DA neurotransmission remains a major pathogenic hypothesis for SZ, transcriptional effects of DA perturbation and the possible differential DA response in a large sample of SZ cases and controls have not been previously investigated. The use of strong system perturbations to increase the size of functional effects of genetic variation (and therefore their detectability) is becoming more recognized^77^. In organisms or even in a simple cellular model, the inherent redundancy of regulatory molecular and cellular mechanisms, e.g., duplicated paralogs^78^, buffers biological systems for the effect of many genetic variations. Such systems’ “robustness” thus may frequently mask the small effect sizes of common genetic variations even if they can cause disease under appropriate environmental stimuli. Our DA perturbation generates an artificial stress situation designed to mimic a relevant environmental stimulus as follows: an asymptomatic individual has a normal range of DA tone, and their DA-related networks only express variation which translates into normal physiological non-pathological states. However, as soon as this individual abuses amphetamine or cocaine (DA-releasing drugs associated with psychosis^79^) or upon exposure to other environmental SZ-risk factors, the activation of cryptic genetic variation may uncover an underlying genetic susceptibility to delusions and hallucinations, and the individual may then develop an episode of psychosis. By using LCLs derived from SZ cases and controls as a simplified albeit imperfect cellular model of SZ, our DA perturbation revealed transcriptomic changes that were invisible to the baseline transcriptomic study, adding additional information for understanding disease biology underlying the genetic contributions to SZ. Compared to genes showing SZ-associated differential expression in baseline or DA-stimulated conditions, genes showing differential DA responses between SZ cases and controls are more enriched for those implicated by SZ-GWAS, CNV studies, and postmortem brain transcriptomic studies (Table 1). Altogether, these results suggest that DA perturbation and testing case-control differential transcriptomic response to DA may add value to studying disease-relevant transcriptomic baseline profiles by enriching for disease-relevant genes.

Genes showing SZ- associated differential response to DA perturbation are highly enriched for those related to immune response (Fig. 3 and Table S4). In part, our results may be enabled by the type of tissue (i.e., immune) used for the experiments. However, we note that genes related to IFN and tumor necrosis factor (TNF) functions are among the top-ranking genes showing SZ-associated differential DA response (Table S3), many of which belong to interferon pathway genes (*IFIT3*, *IFIT2*, *ISG15*, *MX2*, *OASL*, and *USP18*) that have been previously implicated in in blood transcriptome profiles of recurrent major depression^73^. Our results overall are consistent with the immune hypothesis of SZ^80,81^. Maternal immune activation (e.g., via an infectious exposure such as influenza) has been reported to be associated with risk of SZ and autism, and blood levels of cytokines (e.g., IL1B, IL2RA, IL-6, and TNF) are elevated in SZ patients (see reviews^82–84^). Large population-based cohort research also suggests a shared etiology between SZ and several other autoimmune diseases, with increased risks of 1.1–1.6 for SZ^80^. In addition, epidemiological evidence shows a negative correlation between rheumatoid arthritis, an autoimmune disease, and SZ^85^. Most recent SZ GWAS also strongly suggest the involvement of immune mechanisms in SZ pathogenesis. The strongest and most replicable SZ GWAS finding is at the xMHC region^14,17–21^. Furthermore, immune-related genes were enriched among the transcripts differentially expressed by SZ affection status in our previous baseline transcriptome profiling studies^36,37^. Cytokines play roles in cytotoxicity as well as influence DA and other neurotransmission systems that are implicated in the pathophysiology of SZ^23^. For instance, IL-6 modestly increases locomotion in rodents, behaviors modeling hyperdopaminergic-related psychotic symptoms in SZ^86,87^. Because of the possible role of cytokines and inflammatory factors in early-life infectious exposure of SZ patients, anti-inflammatory agents, such as celecoxib and aspirin, have been used as novel treatments in SZ patients to relieve some psychotic symptoms^23^. On the other hand, some antipsychotic drugs rebalance the immune response in SZ patients in microglial cells and astrocytes in the CNS^25,26^. Part of the mechanism is through modulating expression of cyclo-oxygenase-2 (COX-2), and growing evidence from clinical studies with COX-2 inhibitors points to favorable effects of anti-inflammatory therapy in SZ^25,26^.

DA stimulation had a widespread effect on gene expression in LCLs, leading to cell death likely through apoptosis as suggested from our gene set enrichment analysis. Consistently, we only observed the enrichment of apoptosis-related genes in the gene set showing increased expression upon DA stimulation. Apoptotic (and inflammatory) pathways are altered both in brain and the periphery during PD, a disease where neuronal loss is associated with chronic neuroinflammation characterized by microglial activation through the release of inflammatory mediators, as well as apoptosis triggered by the neuronal increase of calcium and DA^59^. Such neurodegenerative aspects (including, e.g., cellular apoptosis/excitotoxicity) have been proposed to confer vulnerability to SZ^25^. Although we did not find robust expression of mRNAs of any DA receptors in LCLs (Table S1), a finding which was supported by the observed inability of DA antagonists to block DA effects on cell growth and gene expression changes (Fig. 2), our main observations appear to be consistent with reported DA effects in brains or in neuronal cell cultures. For instance, four (*HMOX1*, *GDF15*, *AMBP*, and *NQO1*) out of the five most highly DA-responsive genes are related to brain DA function. It has been controversial whether DA receptors are expressed in human peripheral blood lymphocytes^88–92^. Although we did not examine DA receptor expression at a protein level in our LCLs, the observed extremely low level of DA receptor mRNAs suggested our observed DA effects on cell growth and gene expression were likely mediated through non-receptor mediated mechanisms. A possible mechanism for DA function in LCLs may be through DA autoxidation, a process that contributes to DA neuron loss in PD or other neurodegenerative disorders involving DA neurotransmission^15,16^. Like in the human body, DA in cell culture may directly interact with oxygen, yielding quinones plus various free radicals as products^15,16^. In support of this hypothesis, some of our observed highly DA-responsive and brain-function-relevant genes are related to oxidation. For example, the most DA-responsive gene (*HMOX1*) contributes to iron sequestration under oxidative stress^61^, another highly DA-responsive gene (*NQO1*) catalyzes removal of the quinone^69–71^, and *QPRT* (*quinolinate phosphoribosyltransferase*) is one of the three genes within SZ-associated 16p11.2 duplications that responded to DA perturbation. However, the roles of DA autoxidation in SZ pathophysiology remain to be further explored.

It is noteworthy that we have observed a widespread effect of DA on gene expression changes (>90% of the genes) in LCLs and a very high percentage of genes that showed SZ-associated differential expression under baseline (31%) or DA stimulation (21%) conditions. We think that our observation is not a reflection of non-specific effects of a seemingly high DA concentration or other technical artifacts, but rather a result of using a large number of well-controlled LCLs whose authentic biology is revealed by the experiment. First of all, the chosen DA concentration 100 μM is within the range commonly used by the field^56–58^, which is even lower than the estimated DA concentration (1.6 mM) in rat brain synaptic cleft^60^, and did not show much inhibitory effect on cell growth (Fig. 2a). Secondly, to minimize any possible effects of technical confounders, we have intercalated SZ cases and controls in each batch of cell culture, RNA preparation, and RNAseq. Moreover, our QC metrics did not show any systematic case/control bias or batch effects, and we have excluded three potential outlier samples identified by expression PCA and other QC procedures (Figs. S1–3). Instead, our large sample size may have contributed to the large number of differentially expressed genes by boosting the power to detect very small effects of DA on gene expression (only ~150 genes showed > 2-fold expression change upon DA stimulation). Biologically, DA affected cell growth, a central process that involves diverse signaling pathways, and it is thus unsurprising that we observed drastic transcriptomic changes upon DA stimulation. With regards to the large number of genes showing differential expression in SZ cases, our observation may be biologically explained by the association of SZ with immune-related genes including those in the xMHC region^14,17–21^. Indeed, there are 8 HLA genes (*HLA-A*, *HLA-B*, *HLA-DMA*, *HLA-DOA*, *HLA-DOB*, *HLA-DPB1*, *HLA-DRA*, and *HLA-F*) and 21 histone genes at the xMHC region that showed differential expression in SZ cases at baseline (Table S2).

Alternatively, detecting such large number of SZ-associated differentially expressed genes in a well-powered sample may reflect the “omnigenic” model, where most, if not all, genes outside core disease-related pathways are also involved in conferring disease liability by indirectly affecting the functions of core genes^93^. However, we have found that the number of genes showing SZ-associated differential DA response are much fewer than those differentially expressed at baseline or upon DA stimulation (Table 1). This might be explained by the fact that each LCL serves as an internal control when examining the differential DA response, where relevant (but potentially unknown) variables (including confounders) might play much less of a role than at baseline or DA-stimulated conditions.

The use of LCLs as a cellular model vs. brain (presumably the most relevant tissue for SZ) for DA perturbation in our study presents some clear limitations, because some gene expression changes in LCLs substantially differ from that in brain. However, LCLs are the most accessible tissue with a sizable sample, compared with other alternatives such as postmortem brain or neuronal cell lines, and also allow for experimental manipulations such as DA perturbation. Furthermore, a large proportion of gene expression signatures are shared between different tissues^28–35^, and we expect many LCL-expressed genes also show relevant transcription in the brain. Indeed, we have found a nominal enrichment of genes implicated by SZ-GWAS (Table 1), while a large SZ postmortem brain transcriptomic profiling study from the CommonMind Consortium (258 SZ cases and 279 controls) did not find enrichment of SZ-GWAS genes^94^. Another potential limitation of using LCLs as a model is that some functional effects may be an artifact of EBV transformation to produce the LCLs. This concern may be particular relevant for the observation that IFN and TNF pathway genes are among the most significantly SZ-associated differential DA responses (Table S2). However, we have included EBV copy number as a covariate in the analyses (and also excluded monoclonal or pauciclonal LCLs); we thus expect that EBV copy number would pose minimal confounding effects on our observations. Therefore, regardless of LCLs not being brain-derived cells, given the strong support for an immune hypothesis of SZ^95^, our DA perturbation study on LCLs may provide some insights for immune aspects of SZ.

In summary, through DA perturbation that may be pathophysiologically relevant to SZ, we have demonstrated differential transcriptomic effects of DA in SZ cases and controls. Our results yield novel insight into SZ disease biology underlying SZ GWAS and CNV loci, and suggest a new approach to delineate the functional effect of genetic variants of small effect sizes by system perturbation.

## Electronic supplementary material

Supplementary Figure legends

Supplementary Figure S1

Supplementary Figure S2

Supplementary Figure S3

Supplementary Figure S4

Supplementary Figure S5

Supplementary Figure S6

Supplementary Tables
