# Supplementary material for: Transcriptomic signatures of schizophrenia revealed by dopamine perturbation in an ex vivo model
Source: Transl Psychiatry. 2018 Aug 16;8:158. doi: 10.1038/s41398-018-0216-5 (PMC6095865; doi:10.1038/s41398-018-0216-5)
Supplement: Supplementary file 1 — Supplementary Figure legends [file 41398_2018_216_MOESM1_ESM.docx]

**Supplementary Figures**

**Figure S1**. Comparisons of SZ cases and controls who express a gene (i.e., with RPKM>0). (A) and (B) show the sample completion rate (i.e., percentage of cases and controls who have RPKM>0) for a gene at baseline and DA-stimulation conditions, respectively. (C) and (D) show the case (blue) and control (red) comparison of the sample completion rate (i.e., proportion of samples who have RPKM>0) differences in DA and base conditions for all the expressed genes (N= 21,043) and those with larger DA-induced expression changes (>1SD) (N=3,756), respectively. Genes with higher sample completion rate differences tend to have lower expression values.

**Figure S2**. The DA-induced expression changes (fold change-FC) in cases and controls did not show significant bias, i.e., well matched between cases and controls. The scatter plot shows the log2 FC changes of cases and controls for all expressed genes (RPKM>0 in at least 50% of samples under either DA-stimulated or baseline condition.

**Figure S3**. Three samples were defined as outliers by the combined analyses of the mean of sample pairwise gene expression correlation and the principal components analysis (PCA) of gene expression. (A) and (B) show the means of expression correlation of a sample with all other samples at baseline condition and DA stimulation condition, respectively.  Two samples appeared to be outliers (red arrows) at DA stimulation condition. (C) and (D) are representative PCA plots that can most differentiate the outlier samples (red squares) at baseline and DA stimulation conditions, respectively. These contain the same two samples detected as outliers in (B). The 3 outlier samples were excluded from further downstream analyses.

**Figure S4**. Testing statistics are highly correlated between data with or without including cell counts and ATP levels under DA stimulation condition as covariates in linear regression analysis of SZ-associated differential DA response. Pearson correlation (R) between the two is 0.98. –log10 p-values were plotted in the scatter plot.

**Figure S5.** The directions of SZ-associated gene expression differences between baseline and DA-stimulated conditions are highly concordant. Plotted are the beta generated from linear regression analysis of SZ-associated differentia expression at both conditions. The dots highlighted in orange color represented genes that show SZ-associated differential expression at both conditions (n=3,655), of which only one RNA gene ENSG00000278514.1 shows opposite direction of case/control expression difference.

**Figure S6**.  Overlaps between sets of SZ-associated differentially expressed genes. (A) Venn diagram shows very few overlap between SZ-associated differentially expressed genes under baseline and DA stimulation conditions as well as SZ-associated differential DA response in the analysis after regressing out known covariates (Covar) and top 5 principal components (PCs).   (B) Venn diagram shows that most genes that showed SZ-associated differential DA response after regressing out known covariates and top 5PCs also show SZ-associated differential DA response in the analysis when only known covariates were regressed out.
