# Supplementary figures and images for: Transcriptomic signatures of schizophrenia revealed by dopamine perturbation in an ex vivo model
Source: Transl Psychiatry. 2018 Aug 16;8:158. doi: 10.1038/s41398-018-0216-5 (PMC6095865; doi:10.1038/s41398-018-0216-5)

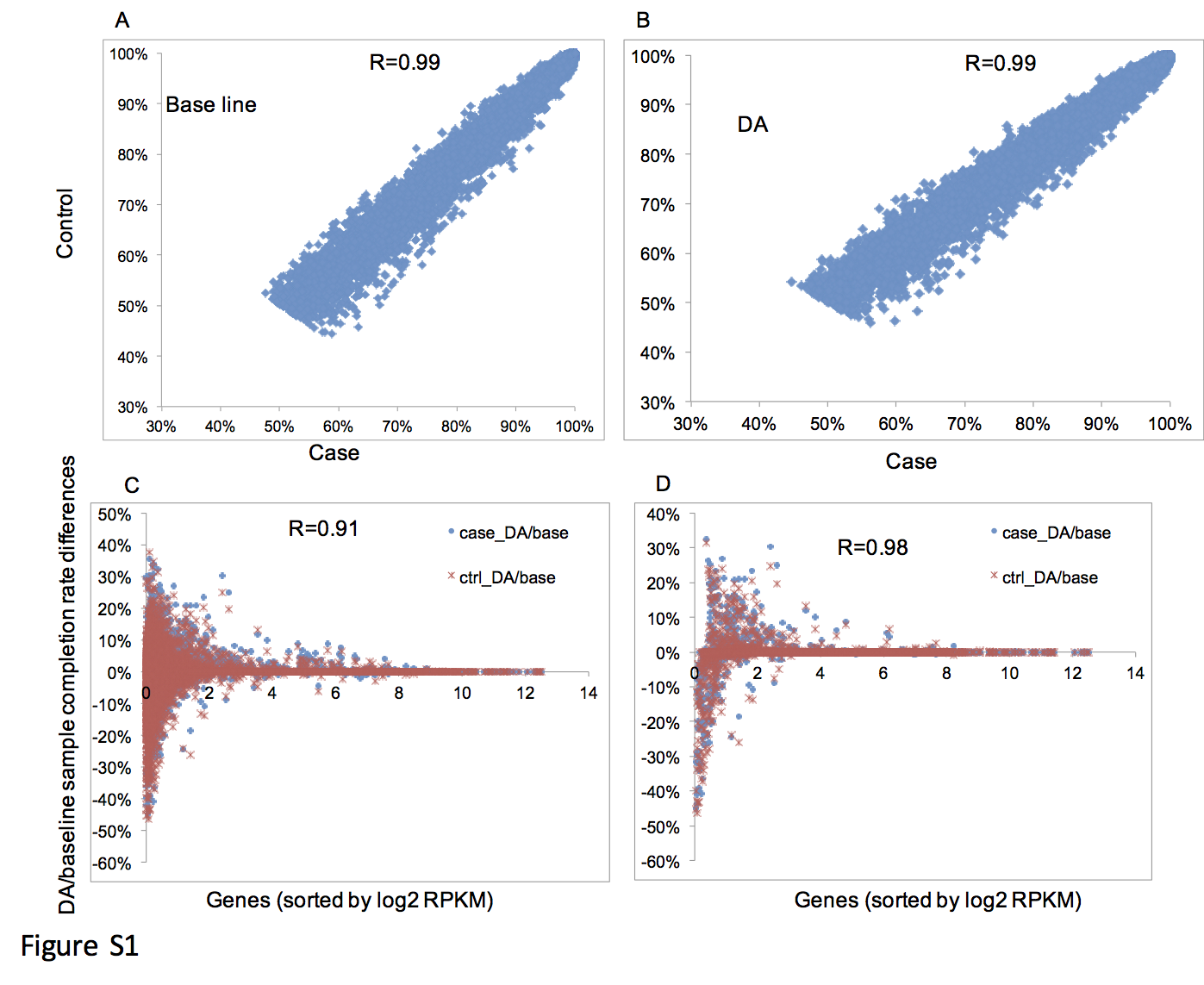

Supplement: Supplementary file 2 — Supplementary Figure S1 [file 41398_2018_216_MOESM2_ESM.tif]

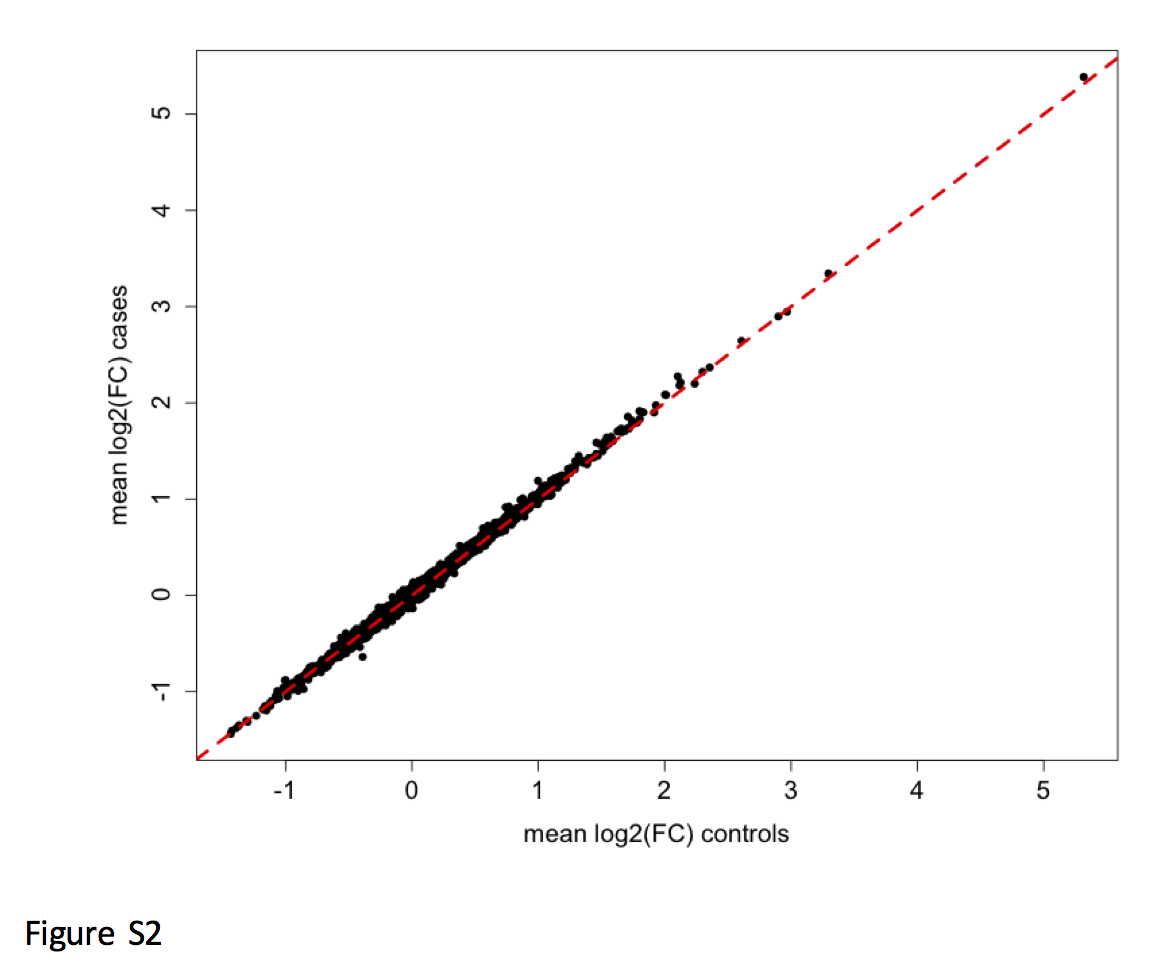

Supplement: Supplementary file 3 — Supplementary Figure S2 [file 41398_2018_216_MOESM3_ESM.tif]

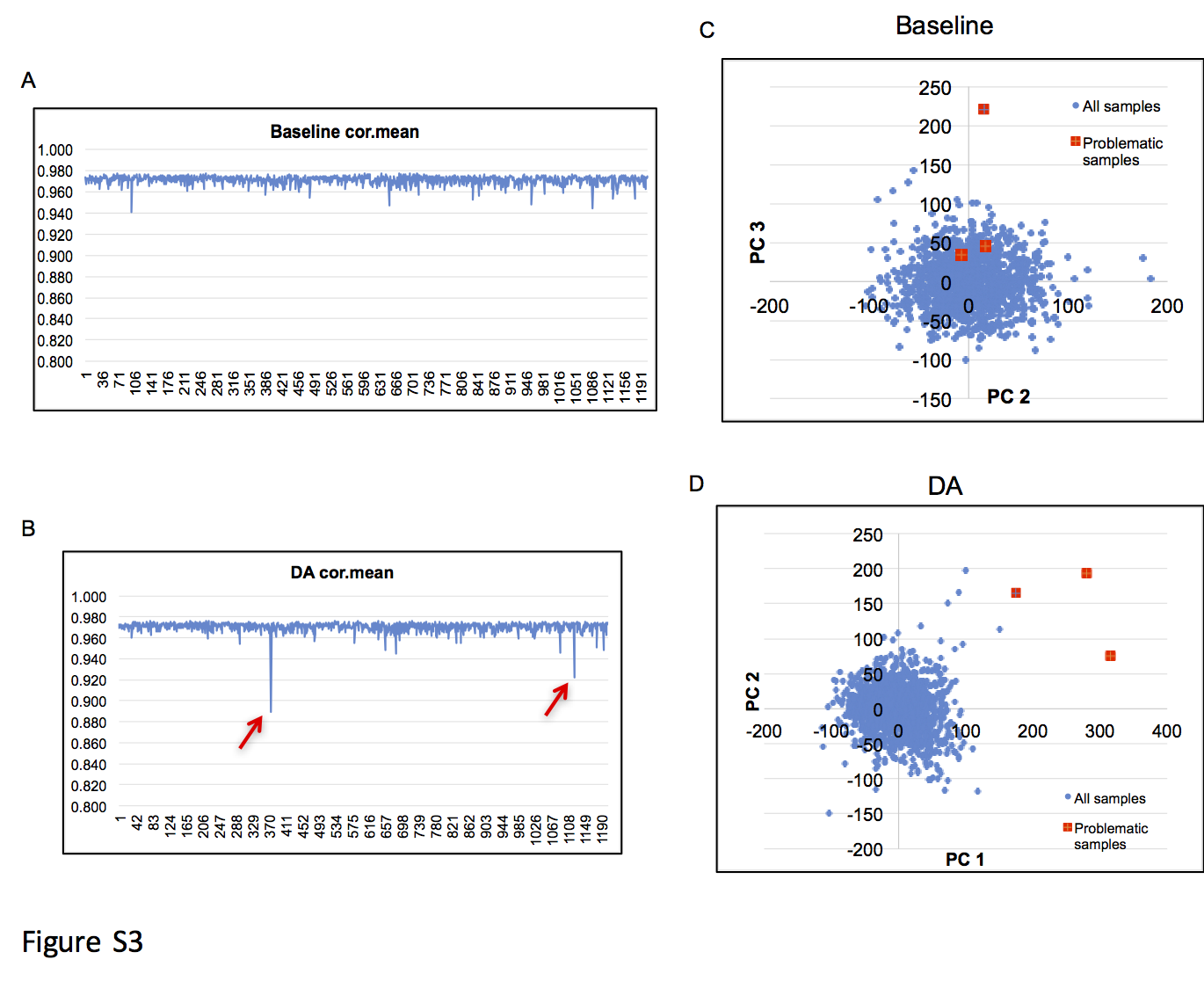

Supplement: Supplementary file 4 — Supplementary Figure S3 [file 41398_2018_216_MOESM4_ESM.tif]

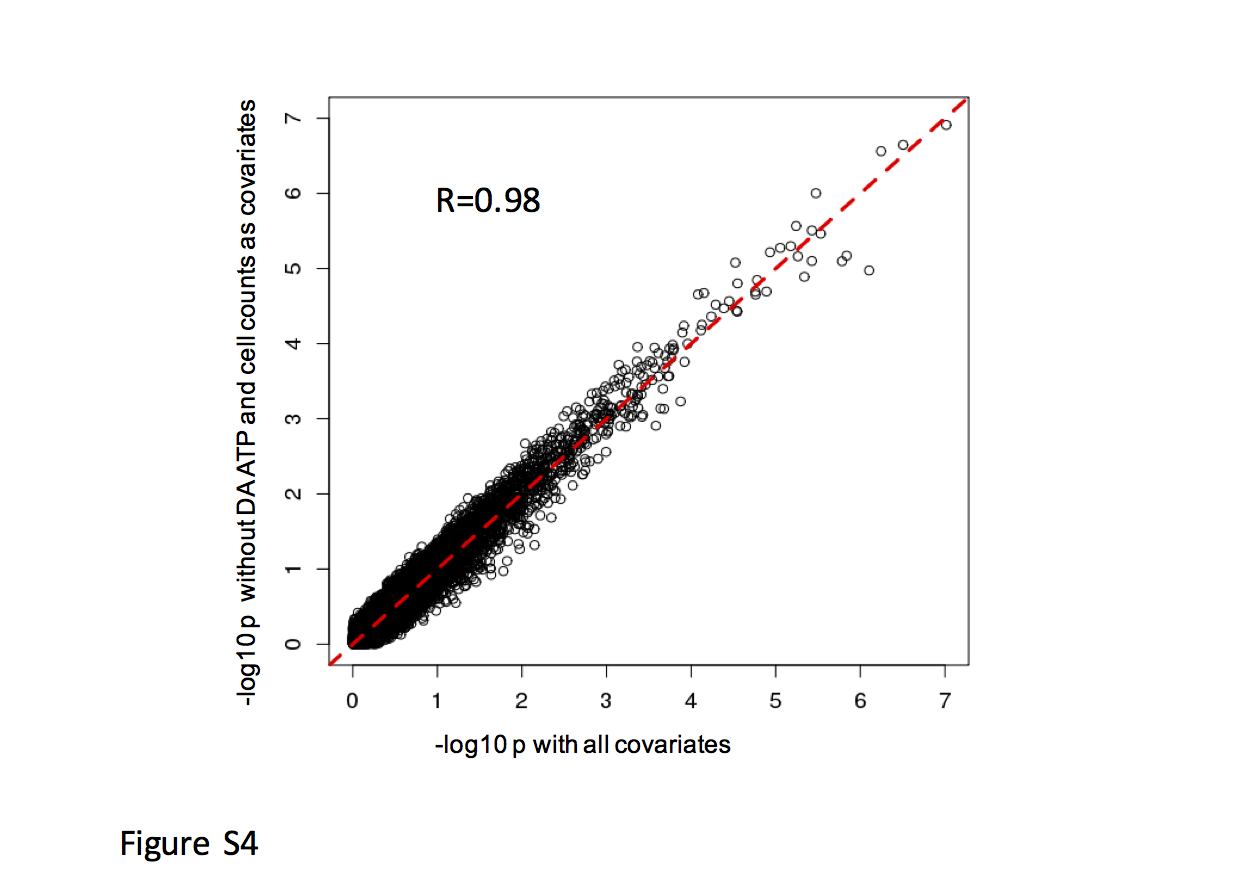

Supplement: Supplementary file 5 — Supplementary Figure S4 [file 41398_2018_216_MOESM5_ESM.tif]

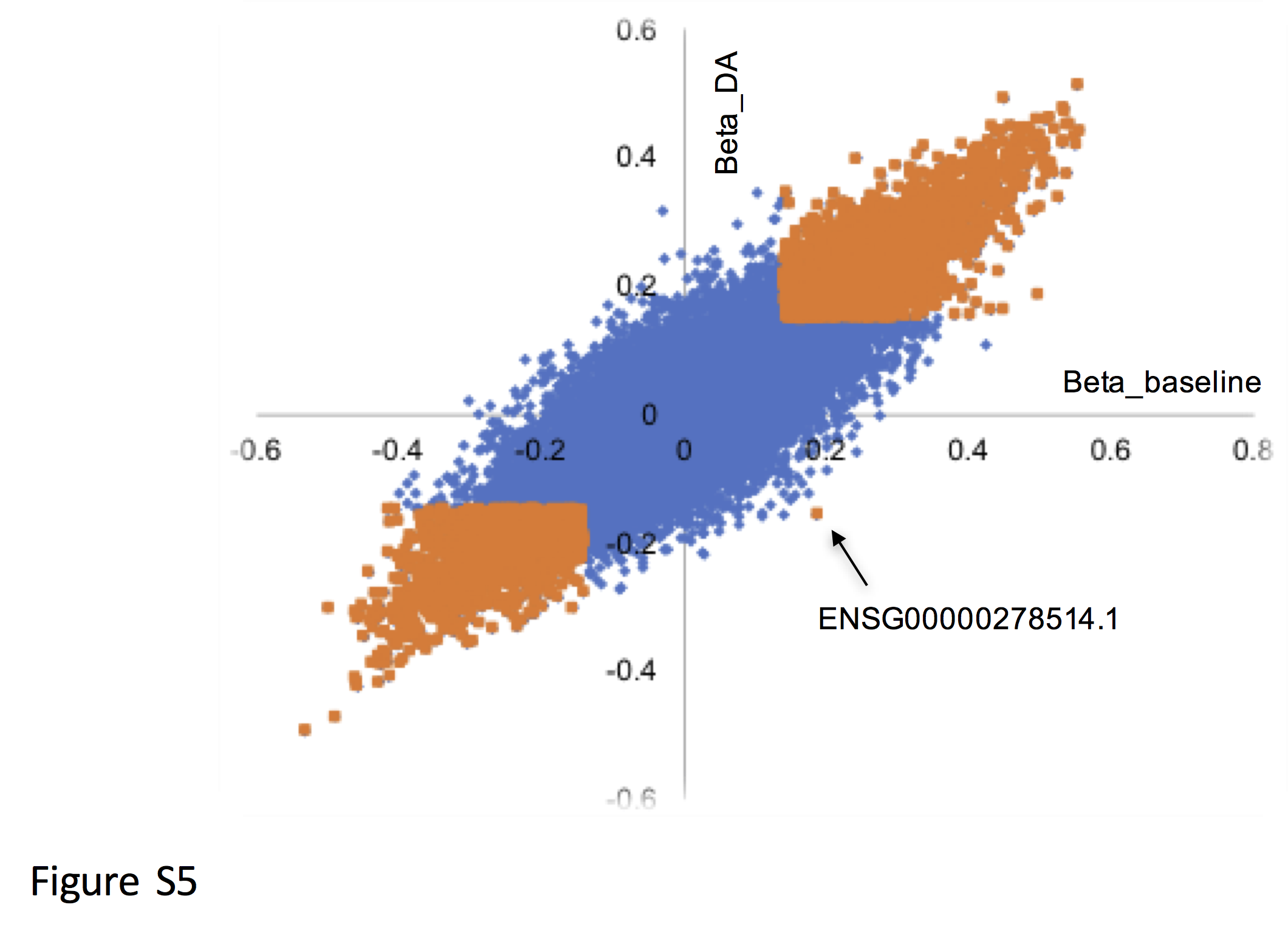

Supplement: Supplementary file 6 — Supplementary Figure S5 [file 41398_2018_216_MOESM6_ESM.tif]

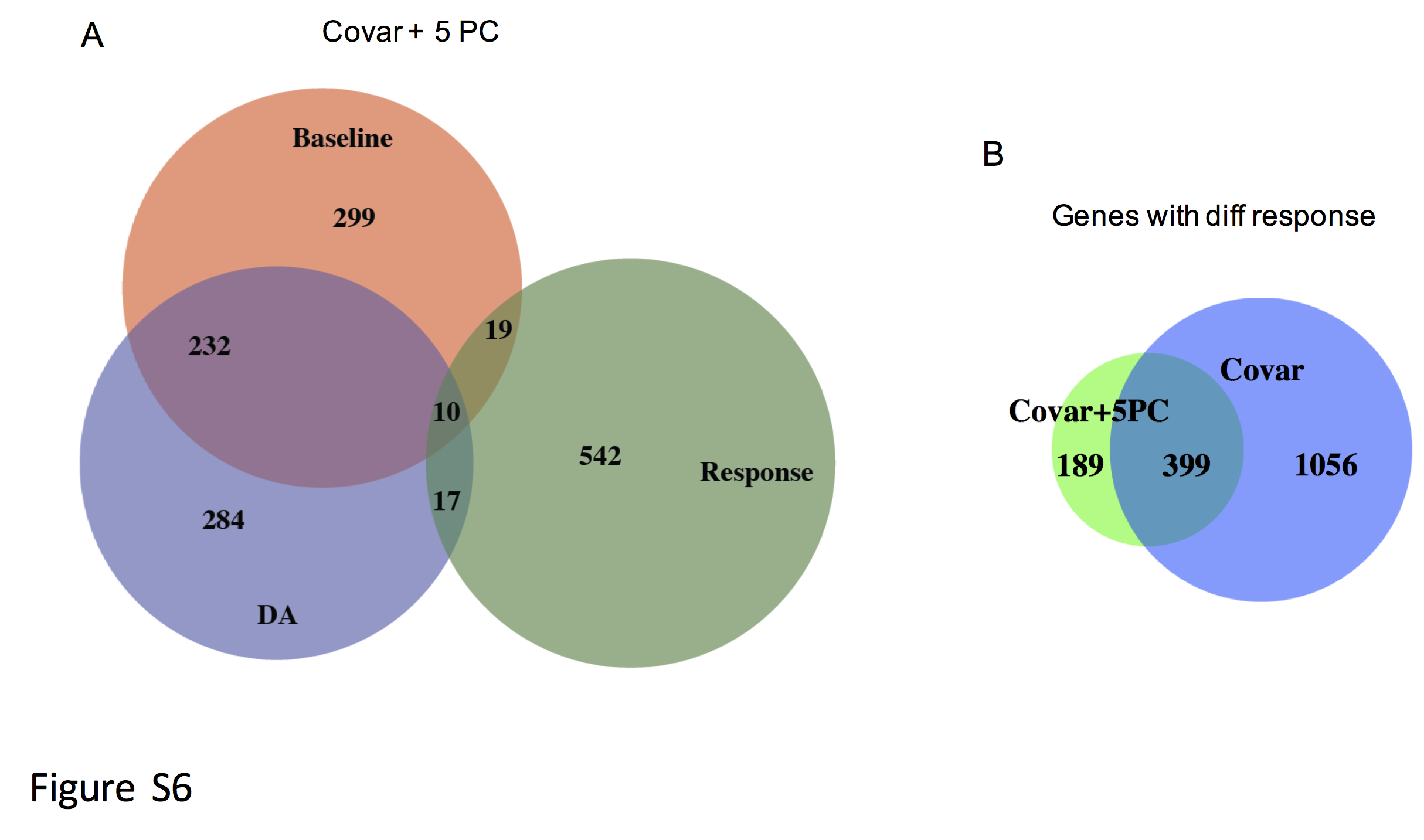

Supplement: Supplementary file 7 — Supplementary Figure S6 [file 41398_2018_216_MOESM7_ESM.tif]
